# Evolutionary and developmental understanding of the spinal accessory nerve

**DOI:** 10.1186/s40851-014-0006-8

**Published:** 2015-01-13

**Authors:** Motoki N Tada, Shigeru Kuratani

**Affiliations:** Evolutionary Morphology Laboratory, RIKEN, 2-2-3 Minatojima-minami, Chuo-ku, Kobe, Hyogo 650-0047 Japan

**Keywords:** Gnathostomes, Neck, Accessory nerve, Trapezius muscle, Development, Evolution

## Abstract

The vertebrate spinal accessory nerve (SAN) innervates the cucullaris muscle, the major muscle of the neck, and is recognized as a synapomorphy that defines living jawed vertebrates. Morphologically, the cucullaris muscle exists between the branchiomeric series of muscles innervated by special visceral efferent neurons and the rostral somitic muscles innervated by general somatic efferent neurons. The category to which the SAN belongs to both developmentally and evolutionarily has long been controversial. To clarify this, we assessed the innervation and cytoarchitecture of the spinal nerve plexus in the lamprey and reviewed studies of SAN in various species of vertebrates and their embryos. We then reconstructed an evolutionary sequence in which phylogenetic changes in developmental neuronal patterning led towards the gnathostome-specific SAN. We hypothesize that the SAN arose as part of a lamprey-like spinal nerve plexus that innervates the cyclostome-type infraoptic muscle, a candidate cucullaris precursor.

## Introduction

**Figure 1:**
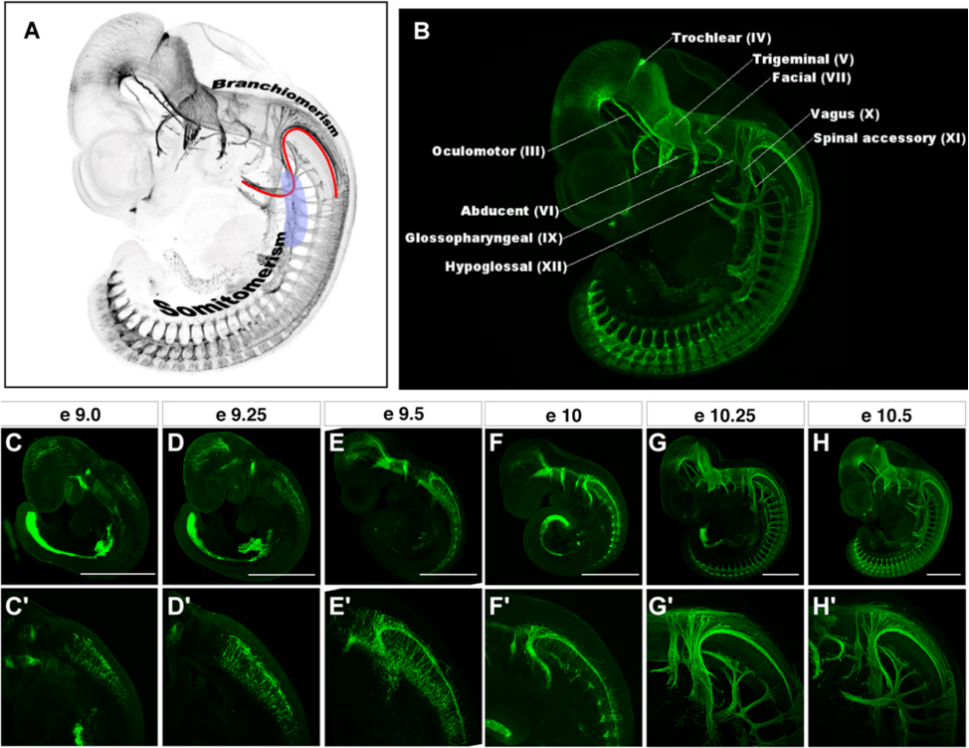
**Immunostained nervous system of developing mouse embryos.** See [4] for the nerve staining method. **(A)** The head–trunk interface is drawn with an S-shaped red line, primarily based on [5]. Light blue indicates the position of cucullaris muscle development. **(B)** The head corresponds to the initial distribution domain of the cephalic crest cells where branchiomeric nerves are predominantly distributed, whereas the trunk domain is coextensive as somites whose derivatives are innervated by spinal nerves. Note that the spinal accessory nerve (XI) issues from the vagus in a close proximity of the interface. **(C- H)** Developmental changes of peripheral nerves of a mouse embryo. **(C’-H’)** Magnification of SAN and vagus nerve of each figure. Bars = 1 mm.

The head of the embryonic vertebrate has a mesodermal component that consists of pharyngeal arch (visceral) mesoderm ventrally and paraxial (somatic) head mesoderm dorsally, giving rise to the branchial muscles and somatic muscles, respectively (reviewed by [1]). The term “branchiomerism” refers to the reiterating patterns of morphological elements associated with or derived from the pharyngeal arches, and “somitomerism” indicates the segmental pattern of somites or somite-associated structures (Figure 1A). This dual segmental pattern of mesoderm characterizes the vertebrate body as a dual metameric organism, as advocated by Romer [2], although it is unknown whether the ancestral form would have possessed a single segmental pattern. The paraxial part of the head mesoderm differentiates into extrinsic eye muscles innervated by cranial nerves III, IV, and VI (Figure 1B), which are classified as general somatic efferent (GSE) nerves, as are the spinal nerves in the trunk, which is defined as the domain that develops segmented somitic mesoderm from which myotomes will arise. The hypoglossal nerve (cranial nerve XII, including occipital nerves in fishes; [3]), which innervates tongue muscles derived from rostral somites, also belongs to this category; the hypoglossal nerve is generally regarded as a bundle of modified spinal ventral roots assimilated secondarily into the head. The ventral part of the head mesoderm, in contrast, gives rise to branchial muscles innervated by branchial cranial motor nerves (cranial nerves V, VII, IX and X), which are classified as special visceral efferent (SVE) nerves (Figure 1B).

These distinct innervation patterns of the peripheral nerves are developmentally prefigured by the distribution patterns of neural crest cells. Cephalic crest cells are specific to the head region, and they migrate along the dorsolateral pathway into the pharyngeal arch, mirroring the morphology of the branchiomeric cranial nerves. In the trunk, neural crest cells migrate in a segmental pattern along the ventrolateral pathway passing somites, where they give rise to dorsal root ganglia. Thus, the distribution pattern of neural crest cells reflects the head/trunk organization [5]. However, the postotic crest cells use both pathways of neural crest migration.

The head and trunk are not simply juxtaposed onto each other anteroposteriorly, but the pharyngeal arches and rostral somites co-exist in the postotic region; the boundary of these two components forms an S-shaped head/trunk interface (Figure 1A) that is conserved in all vertebrate species [5]. This interface develops into the future ‘neck’ region of some vertebrates [6], corresponding to a region that develops a set of intermediate structures, partly reflecting head-specific features and partly trunk, like the neck muscles that are derived from somites and associated with crest-derived connective tissues (reviewed by [5]). The cucullaris, tongue, and infrahyoid muscle complexes, as well as the nerves that innervate them (cranial nerves XI and XII), can be counted among these structures (Figure 1A). The morphological and evolutionary evaluation of the cucullaris muscle (which became the trapezius and sternocleidomastoid muscles in mammals; [7]) and the spinal accessory nerve (SAN) that innervates it have long been controversial, primarily because these structures arise in the interface between the head and trunk domains (Figure 1A; see [8]).

Although a large number of studies have examined the accessory nerve (cranial nerve XI), this nerve cannot be easily classified into this standardized SVE and GSE scheme of the vertebrate body, an issue that has confused many researchers [9-11]. Anatomically, the accessory nerve is usually thought to consist of two components, the cranial accessory nerve, which innervates the pharynx and larynx muscles, and the SAN, which innervates the neck muscles, including the trapezius and sternocleidomastoid muscles (for cortical input to the SAN, see [11]). Recently, Benninger and McNeil reviewed papers of anatomical, embryological, and molecular studies to propose that the SAN is a transitional nerve that does not belong either to GSE nor SVE [12]. The current review focuses on the evolution of the SAN and in particular on the question of whether it belongs to SVE, GSE or another category of efferent nerves in the comparative anatomical, embryological, and evolutionary developmental (evo-devo) contexts. We reconstruct the evolutionary sequence to explain the evolutionary origin and acquisition of the SAN.

## On the developmental origin of the cucullaris muscle

Phylogenetically, the sternocleidomastoid and trapezius muscles arose from a single muscle, the cucullaris, which split into two parts during mammalian evolution. Because the cranial accessory nerve branch joins the vagus nerve branch and innervates palatal, pharynx and larynx muscles that derived from the branchial arch, these cranial components are considered to be SVE, a genuine part of the vagus (Xth nerve). Because the SAN exclusively innervates the cucullaris muscle, whose developmental origins remain controversial, the nature of the SAN has also been controversial.

There are several different theories to explain the developmental origin of cucullaris muscles. For example, the cucullaris muscle has been reported to arise from branchial arch mesoderm [13] or somites [14-17]. However, a new interpretation based on a fate-mapping study in chick-quail chimeras is that most (90%) of the cucullaris muscle is derived from the lateral plate in chicken embryo [18].

Although the origin of the cucullaris muscle remains controversial, the innervation by cervical spinal nerves in some vertebrates suggests that the cucullaris muscle does have some features specific to somatic components (see below and [19]). The innervation patterns differ among species in terms of the proportions innervated by SAN and the cervical spinal motor nerves. For example, some ungulates (giraffes, camels, and llamas) either have no SAN or have only a rudimentary SAN, and instead the muscles are exclusively innervated by the cervical spinal nerves [10,20,21]. Although these studies were conducted by different methods in a different era, it seems clear that somatic nature was constantly detected in the SAN. Thus, it is plausible that the SAN would be, at least in part, a somatic component, despite its being considered by some anatomists to be a branch of the vagus nerve, and therefore SVE. This drives us to the question of which category the SAN belongs to in terms of developmental and evolutionary contexts. Thus, to better understand the nature of the SAN, the location of its nucleus, its cytoarchitecture and its neural progenitor domain, as well as developmental and embryological analyses in the context of evo-devo, are extremely important.

Located between the head and trunk regions, the cucullaris muscle is one of the major components in the vertebrate neck [6] (Figure 1A), and together with the SAN, it is regarded as one of the synapomorphies that define gnathostomes, as these structures are both found only in gnathostomes and not in cyclostomes. Although the trunk and head muscles are known to develop differently from each other, the morphological and developmental nature of the cucullaris muscle has long been a subject of controversy [22,23]. Edgeworth [13] believed that the neck muscles are derived from branchial arches, and recent comparative morphological and experimental analyses have come to a similar conclusion [18,24]. In particular, Theis and others have found in very careful experiments that the cucullaris muscles of chicken and mouse develop by deploying the head myogenic program, and the majority (90%) of the cucullaris is derived from the lateral plate mesoderm at the occipital level, although it remains unknown whether this mesoderm also differentiates into pharyngeal arch mesoderm [18]. On the other hand, several reports based on histological observations and chick–quail grafting experiments have indicated that this muscle arises from rostral somites [14-17,25], indicating that the cucullaris muscle should be regarded as a somitic muscle like typical trunk skeletal muscles. Further evidence to support this is available from analyses using the axolotl, in which the muscles arise from rostral somites in a manner very similar to that in avian embryos [26]. Thus, the origin of the cucullaris muscle remains a matter of controversy, and further experimental analyses are needed to explain the inconsistent results obtained from the previous studies.

The question of the developmental origins of connective tissues (the tendons and fascia) for the cucullaris muscles is also an important issue because, in the chicken, these tissues for the extra-ocular and branchial muscles are derived from neural crest cells [16], and those for trunk muscles are derived from mesodermal (somites and lateral plate) cells [27,28]. The connective tissues for the cucullaris muscles are generally believed to be of neural crest origin (in the chicken), similar to those for the extra-ocular and branchial arch muscles. In the mouse as well, these tissues are derived from neural crest cells [6]. However, recent studies have suggested that in anamniotes (zebrafish and axolotl), the connective tissue of the cucullaris muscle is of mesodermal origin [29,30].

One possible explanation for the discrepancy regarding the origin of the cucullaris muscle is that the embryos used in previous studies were at different developmental stages. As has been pointed out [31], mapping data may differ depending on the timing of mesodermal specification (see [16,32]). Noden [16] has shown that rostral somites in a stage 10 chicken embryo differentiate into pharyngeal arch muscles innervated by the vagus nerve, a nerve that innervates the branchiomeric elements, not the trunk. It has also been shown that the paraxial and lateral domains of the head mesoderm only become specified secondarily in a later stage of development in shark embryos, as assessed by expression of *Pitx2* and *Tbx1*, markers for the paraxial and pharyngeal arch markers, respectively [33] (Adachi et al., 2012).

Thus, cucullaris muscles exhibit inconsistent developmental and embryological signatures, and even if that is partly ascribed to technical differences in experiments, the developmental patterning of this muscle appears to involve extremely complex patterns and processes that standard experiments are inadequate to elucidate. It is also true that the muscle arises in a very peculiar part of the vertebrate body, corresponding to the head–trunk interface or the ‘neck’ by the definition of Matsuoka et al. [6], where both the cephalic (pharyngeal arch elements and cephalic crest cells) and typical trunk (somitic derivatives and lateral plate) coexist. The same difficulty is associated with the accessory nerve. As shown in Figure 1C-H, cell bodies of the hypoglossal nerve, vagus nerve and SAN cannot be distinguished initially among the neuroblasts in the ventral hindbrain (Figure 1C). The hypoglossal neurons (GSE) start to grow axons ventrally and those of the vagus nerve (SVE) and SAN elongate axons dorsally and extend along the head/trunk interface on e9.0 (Figure 1C). The latter axons are fasciculated during later development. Thus the GSE and SVE become distinct from each other secondarily in development (Figure 1C-H).

## Localization of the SAN nucleus in vertebrates

The location of the nucleus and axon tract of the SAN have been investigated in several taxa of animals, but not much consideration has been given to the evolutionary origin of the SAN, except for a description of the cranial and first spinal motor nuclei and root [34]. The SAN nucleus is located in the dorsolateral part of the ventral horn in the medulla and spinal cord in mammals, but the location is variable among other vertebrates.

**Figure 2:**
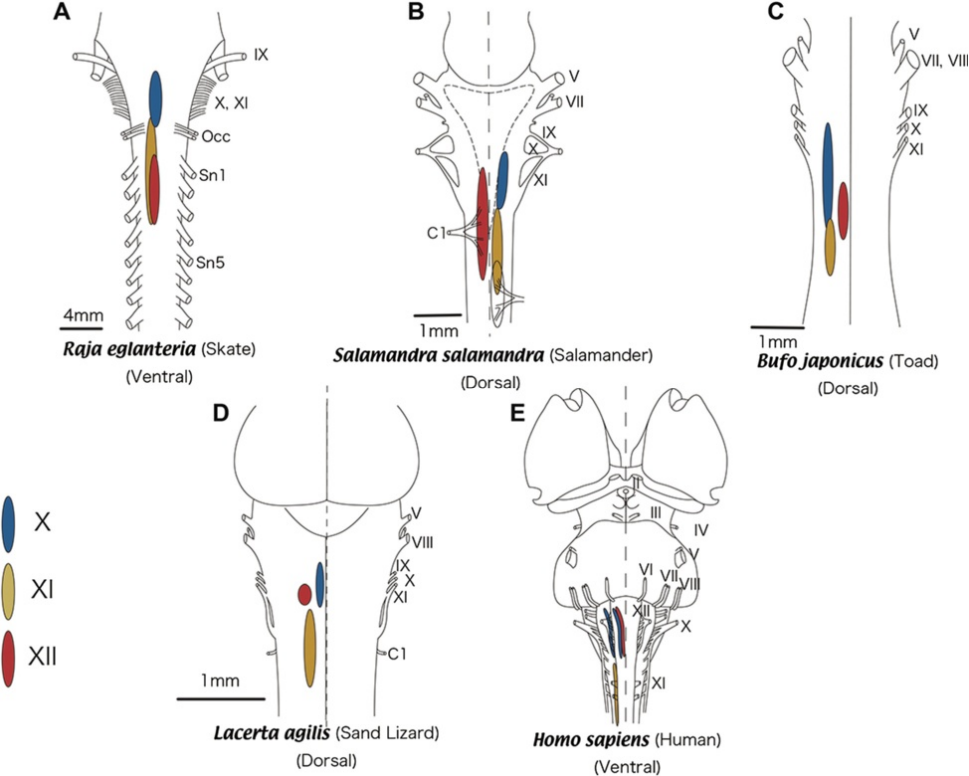
**Comparison of locations of the vagus (X), spinal accessory (XI), and hypoglossal (XII) motor nuclei.**

An anatomical description of the SAN has been given in a representative elasmobranch, the skate *Raja eglanteria*. Although the axonal tract of the SAN sprouts dorsally and is intermingled with the vagus branch, the SAN nucleus is clearly separated from that of the vagus nerve and forms a caudoventral motor nucleus located in the same plane as that of the ventral spinal motor neurons [35]. These authors note that the ancestral gnathostomes would also have possessed an accessory nucleus located in the rostral spinal cord and probably isolated from any of the branchiomeric nerve components, including the vagus [36]. The rostrocaudal level of the SAN nucleus overlaps that of the spinal motor column, with its rostral limit extending slightly rostral to that of the spinal motor neurons (Figure 2A), indicating that the SAN nucleus lies rostral to that of the XIIth nerve (Figure 2A).

In urodeles, Wake et al. [37] studied the distribution of the SAN nuclei by using HRP techniques in 22 species of salamanders. In all of the species, axons of the SAN leave the brainstem with fibers of the Xth nerve as they do in other gnathostomes, and the nuclei are localized in the ventromedial and lateral parts of the spinal cord. Along the anteroposterior axis, they extend from the obex to the caudal end of the nucleus of the third spinal nerve (Figure 2B). The anuran SAN nucleus has been studied in the Japanese toad (*Bufo japonicus*) by retrograde labeling with cobaltic lysine complex [38]. In this animal, the SAN innervates the interscapularis and cucullaris muscles [39] verifying the homology between the anuran SAN and the SAN in other gnathostome species. Although the SAN of the anuran arises as a branch of the Xth cranial nerve trunk, as in other vertebrates, the ventrolateral positioning of somata resembles that of the ventrolateral group of the XIIth cranial nerve (XIIthVL) and *ramus thoracicus superior anterior* of the second spinal nerve (TSA-MNs). Its rostrocaudal distribution mostly overlaps with that of the XIIthVL and TSA-MNs that form the spinal motor neuron column of the second spinal nerve. The rostrocaudal level of the SAN nucleus overlaps that of the first spinal nerve nucleus, but its rostral limit is caudal to that of the hypoglossal nerve (Figure 2C).

In addition to the similarity in the locations of the nuclei, the perikarya of the SAN morphologically resemble those of the hypoglossal motoneurons, which are significantly larger than those of the vagal motoneurons. Furthermore, the dendritic configuration of the SAN is similar to the lateral dendritic array of the spinal motor neurons, which is distinctly different from that of the branchial motor neurons (Vth, VIIth, IXth and Xth cranial motor neurons; [38]): the SAN primary dendrites are oriented along the dorsolateral–ventromedial axis, whereas those of the branchiomeric nerves are oriented along the dorsomedial–ventrolateral axis.

In reptiles, the nucleus of the SAN has been identified in an adult lizard (*Lacerta agilis*) by retrograde labeling [36]. The nucleus is located caudal to that of the XIIth and Xth nerves in the rostrocaudal dimension and intermediate between the Xth nerve and the spinal motor neurons in the dorsoventral dimension (Figure 2D). Similar to those in amphibians, the spindle-shaped perikarya have dorsolateral–ventromedial dendritic arborizations. Again, this dendritic configuration is similar to that of the lateral dendritic array of the spinal motoneurons in lizard. In addition, the perikarya of these motor neurons are also significantly larger than those of the largest vagal neurons.

The cytoarchitecture of SAN neurons is very similar among the skate, amphibians, and lizards (Figure 2A–D). A recent study has also shown that the SAN of some teleosts has a similar morphological configuration [40]. All these reports suggest that this pattern does not reflect a secondarily shifted pattern, but rather an ancestral state of the SAN in gnathostomes. Moreover, the cytoarchitectural differences between the SAN and Xth nerve are inconsistent with the idea that the Xth nerve is the phylogenetic origin of the SAN, as has been proposed [22]. These cytoarchitectural differences rather imply that the evolutionary origin of SAN is independent of the vagus nerve, and the axonal route came secondarily to accompany that of the vagus nerve.

Mammalian SAN nuclei have been localized in adults of many animal species, based on retrograde labeling with a variety of tracers (HRP, DiI, fluorescent tracers or cobalt placed in the cucullaris muscle or directly into the SAN; [14,22,41-65]. The results of some of the reports differ—in a few cases, even for the same animal species by using similar retrograde labeling methods. For example, SAN nuclei have been identified in the caudal part of the medulla and the rostral part of the spinal cord in sheep [47], monkey [66], and rabbit [64], whereas other researchers located them in the rostral part of the spinal cord in cat [67], monkey [60], and sheep [68]. Moreover, disagreement also exists as to the number of cell columns of the SAN longitudinal nuclei. The above discrepancy may be ascribed, at least in part, to the difference in accuracy of observation derived from different labeling methods. Recently, Ullah et al. [69] reported that there are three longitudinal columns that innervate the sternocleidomastoid muscle in addition to one trapezius column in the adult rat [69], whereas Flieger [47] and Clavenzani et al. [68] found two groups of longitudinal nuclei for the sheep SAN. Hayakawa et al. [63] reported that there are two columns, one each for the sternocleidomastoid and trapezius muscles. Finally, a single longitudinal column was identified in the rat by retrograde labeling of the SAN branches with DiI [65], and similarly in the cat [51,70] and baboon [59], only one column of somata in the spinal cord has been reported.

Similar to the differences in findings regarding the number of columns, results for the cell population of the mammalian SAN have varied. For example, there are two SAN cell populations in the adult rat as found by retrograde labeling of SAN axons [65]. These authors clarified the localization of motor neurons, which extended axons either directly through the SAN or through the ventral rami of the C2–C6 cervical nerves to innervate the trapezius. The somata of trapezius-innervating neurons whose axons grow through the spinal accessory nerve are larger than those of neurons that pass through the cervical nerves. Yan et al. [65] presumed that the neurons that extend axons through the SAN are mainly alpha-motoneurons (and some gamma-motoneurons) and that the axons through the cervical nerves are mainly gamma-motoneurons (and some alpha-motoneurons). The axons that travel through the cervical nerves exclusively innervate one or more of the “intrafusal” muscle fibers that are found only within the muscle spindle stretch receptors. These results indicate that the motor input to the trapezius would be conveyed mainly via the SAN in the rat. Consistent with this report, some researchers found that the cervical spinal nerves exclusively contribute proprioceptive fibers [22]. The recent consensus is that the rat SAN nucleus is composed of medial and lateral columns. The medial column, which innervates the sternomastoid and cleidomastoid muscles, is located at the dorsomedial edge of the ventral horn at the level of C1 to C3, and the lateral column, which innervates the trapezius and cleido-occipital muscles, is located in the dorsolateral part of the ventral horn at the level of C2 to C6 [22,63,71] (Matesz and Szekely 1983; Krammer et al., 1987; Hayakawa et al., 2002). In avians, on the other hand, the spinal motor axons that innervate the cucullaris sprout from dorsal roots, and these neurons exhibit branchiomotor properties by expression of *Phox2b* and *Isl1* [19,72,73].

From the above, we can infer the general phylogenetic changes in the positions of nuclei for the Xth and XIIth cranial nerves and SAN. One obvious tendency in the lineage towards the mammals is that the XIIth nerve nucleus migrated rostrally to reach as far as the dorsal Xth nerve nucleus, as has been noted by Kappers [74,75]. Kappers had proposed a hypothesis, known as the ‘neurobiotaxis’ theory, that cell bodies tend to migrate in evolution towards the source of the stimuli that their axons receive most frequently, and thus he postulated that the gustatory and tactile sensibilities of the mouth cavity drew the XIIth nerve nucleus up toward the mouth [34,75].

**Figure 3:**
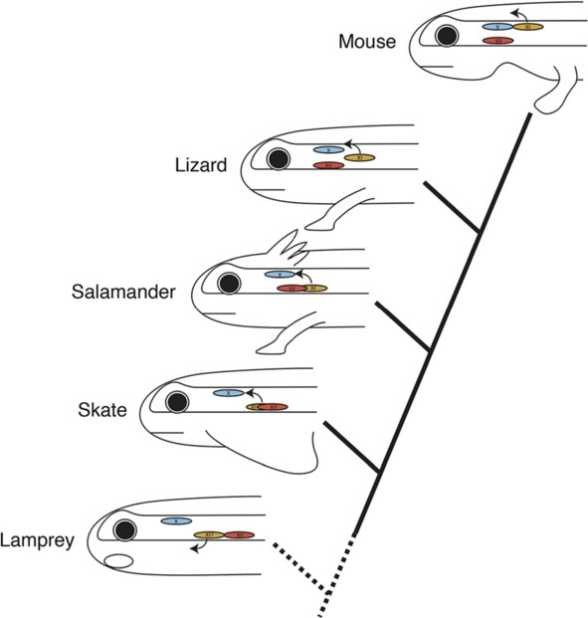
**Hypothetical scenario of the evolution of the accessory nerve toward mammals.** Motor nuclei are colored blue for vagus, orange for SAN, and red for the hypoglossal. Arrows indicate the direction of the intramedullar axonal growth to sprout from the brain stem.

The observation of morphological differences between the SVE and the SAN supports the GSE nature of the SAN. As noted above, the diameters of the SAN axons are larger than those of the Xth nerve: although the pharyngeal branches of the Xth nerve consist predominantly of intermediate and small-diameter myelinated axons, the SAN consists predominantly of myelinated axons of conspicuously larger diameter [35]. Furthermore, the morphological features of the central nervous system–peripheral nervous system (CNS–PNS) transitional zone (TZ) of the SAN is similar to that of the spinal motor rootlet and different from that of the vagus nerve [76,77]. The TZ is a segment of a nerve root or rootlet that contains both central and peripheral nervous tissue [78]. Central to this TZ, myelin sheaths of nerves are formed by oligodendrocytes and the supporting tissue is astrocytic. On the peripheral side, sheaths are formed by Schwann cells that are enveloped in endoneurium. The transitional node represents a hybrid between the central and peripheral nodes [79]. Although the SAN rootlets are located at a position between the ventral and dorsal cervical rootlets, their morphology closely resembles the ventral rootlets, consistent with their composition of primarily motor fibers [76]. This clearly illustrates the difference between the Xth nerve and SAN, as well as the similarity between the spinal motor nerve and SAN. Thus, through comparison of the SAN, Xth, and XIIth nuclei on the phylogenetic framework of jawed vertebrates, it can be inferred that the SAN developed in the ventral spinal plane in the ancestral condition, but whether it was positioned at the levels of the Xth and XIIth nuclei, as in the skate, or not, as in osteichthyans, remains undetermined (Figure 3). To solve this problem, we turned our attention to an outgroup taxon, the lamprey.

## Evolutionary origin of the SAN

**Figure 4:**
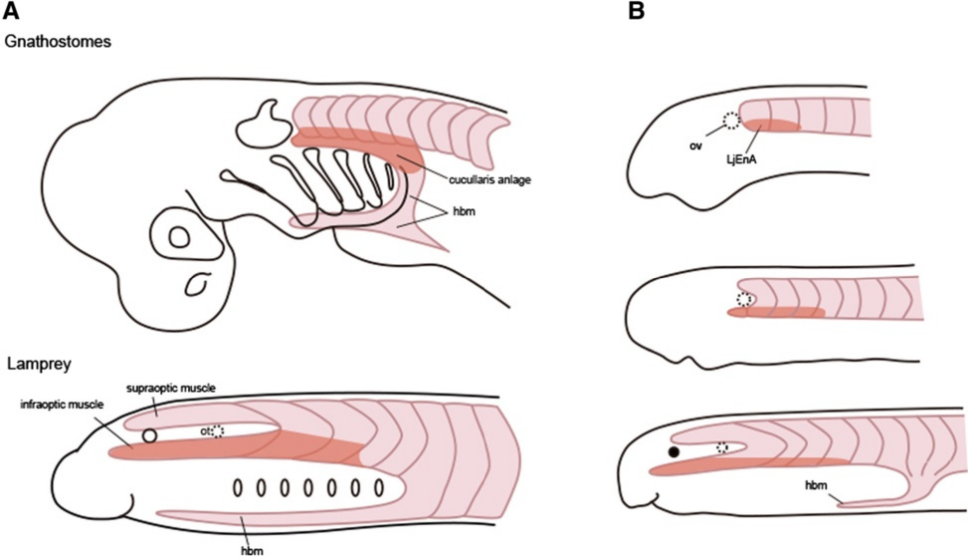
**Schematic illustration to show the evolution of the cucullaris muscle.** Based on the assumption that the cucullaris first arose through modification of rostral somitic muscle. **(A)** Comparison of gnathostomes (top) and ammocoete larva of the lamprey (bottom). Note that the infraoptic muscle of the lamprey occupies identical topographical position as that of the cucullaris (hypothetical somitic components) in gnathostomes. **(B)** Developmental sequence of the infraoptic muscle in the lamprey from Tahara's stages 24 to 28. This muscle is apparently specified by *En* (*LjEnA*) expression, which is restricted in ventral moieties of lamprey myotomes. hbm, hypobranchial muscles; ov, otic vesicle. Modified from [82].

**Figure 5:**
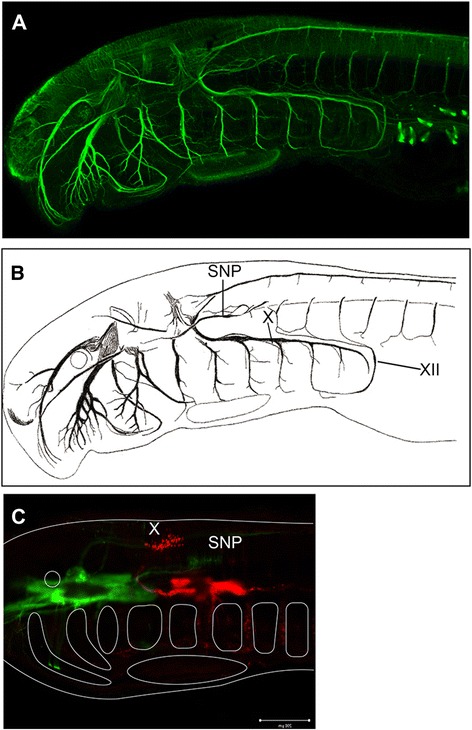
**Hypothetical precursor of the SAN in the lamprey. (A)** Nerve fibers of a larval lamprey, *Lethenteron japonicum* (Tahara's stage 25; see [86]) visualized by whole-mount immunostaining using anti-acetylated tubulin monoclonal antibody (Sigma). See [87] for staining procedure. **(B)** Illustration of the same specimen as in **A** to show the locations of SNP, vagus nerve (X) and the hypoglossal nerve (XII). **(C)** Locations of the spinal motoneurons innervating infraoptic muscles in the ammocoete larva of the lamprey. To label the neurons, rhodamine- and fluorescein-conjugated dextrans (Sigma, St Louis, MO) were injected into the posterior pharyngeal arches and infraoptic muscle, respectively, of a Tahara's stage 25 larva [86], according to the method described by [88]. The injected embryos were incubated at room temperature for 30 minutes before fixation. The fixed specimens were dehydrated and clarified with a 1:2 mixture of benzyl alcohol and benzyl benzoate (BABB). Note that the cell bodies of the SNP are located caudal to those of vagal motoneurons, where the ancestral SAN is expected.

Living agnathans are thought to represent the most basal vertebrate lineage, which diverged from the rest of vertebrates more than 500 million years ago [80]. The SAN and cucullaris muscle that we see in gnathostomes are apparently absent [81]; therefore, the cyclostomes potentially exhibit a morphological state prior to the acquisition of SAN. In the gnathostomes, the precursors of the cucullaris muscle migrate a long distance caudally (Figure 4; reviewed by [82]), as migratory muscle precursors (MMPs) do [83,84], to differentiate into specific hypaxial muscles, including those of the limbs, tongue, and diaphragm. The MMPs migrate in response to the expression of specific genes (the *SF/HGF* and *c-Met* signaling pathway, *Pax3* and *Lbx1*). Although the lamprey lacks morphologically typical MMPs with de-epithelialization, the rostral two somites express homologs of *Pax3* and *Lbx1*, which control the de-epithelialization and long-range migration of the MMPs to give rise to the inferior and superior optic muscles (Figure 4; IOM and SOM; [8,82]). The embryonic configuration of the IOM and SOM in the lamprey is reminiscent of that of the neck muscles, namely the cucullaris and its antagonist, in the stem gnathostomes (placoderms; [85]). Our unpublished observation based on retrograde labeling indicated that the IOM is likely innervated by the spinal nerve plexus (SNP), a lamprey-specific spinal nerve located caudal to the Xth nerve and rostral to the XIIth nerve (Figure 5). Although the SNP axon sprouting from the CNS is not clearly segmented nor organized like a typical spinal motor nerve, the labeled somata in the lamprey were localized in a ventromedial position similar to that of the spinal motor neurons of this animal (Figure 5). As mentioned above, the relative rostrocaudal positions of the gnathostome SAN and XIIth nerve have gradually changed during evolution. Extrapolating from this, it is plausible that the lamprey SNP corresponds to the position expected for the hypothetical ancestral SAN, thereby suggesting that the SAN was ancestrally positioned at the levels of the Xth and XIIth nuclei (Figure 3). This speculation is also consistent with the evidence that the gnathostome SAN has many somatic spinal nerve cytoarchitectural features, and as Sperry and Boord [35] have described, the SNP is located in the rostral spinal cord, unrelated to any of the components of the vagus or the branchial arches.

Regarding the hypothetical GSE origin of the SAN, the next question to be asked is why the SAN axons sprout dorsolaterally from the medulla. One explanation for this is provided by the phenotype of the *Lhx3/Lhx4* knockout mouse, in which the axons from the cervical spinal motor nerves sprout dorsolaterally rather than ventrally, reminiscent of the pattern found in the SAN. After exiting from spinal cord, the axons of the *Lhx3/Lhx4* knockout mouse form a fascicle between the neural tube and dorsal root ganglia that extends rostrally like the axons of SAN motor neurons [89]. Thus, the change in axonal sprouting is apparently determined by the expression of one or two genes during development, and possibly through evolution as well.

The above hypothesis describes how the rostral spinal motor nerves could have changed into the SAN during vertebrate evolution. The ancestral vertebrate would probably have had a precursor of the SAN between the Xth and XIIth nerve. Due to a rostral shift of the XIIth nerve, the rostrocaudal level of the XIIth nerve nucleus overlapped that of the SAN in the ventral spinal cord (the SAN nucleus lies lateral to the XIIth nucleus), and the SAN axons began sprouting dorsally, as seen in the skate (Figure 3). Thus, the SANs are now found caudal to the XIIth nerve in amphibians, lizards, and mice. In mammals, the nucleus of the SAN is elongated caudally, possibly due to the effect of ‘neurobiotaxis’ (*sensu* Kappers), as stimulation comes from connections with the rostral cervical spinal nerves [22]. Collectively, the phylogenetic changes of the relationship between the positions of the Xth, XIIth, and SAN nuclei could be as described in Figure 3.

## Molecular bases for the development of the SAN

**Figure 6:**
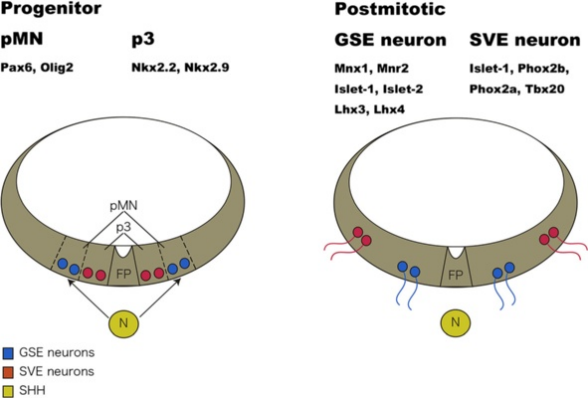
**Schematic illustration of the developmental process.** GSE and SVE neurons are derived from pMN and p3 domains, respectively. These domains are specified by expressions of different sets of regulatory genes. Note that the position of SVE shifts dorsolaterally during development. Modified from [92].

The transcription factors involved in development of the spinal cord and hindbrain motor neurons have been well studied. In the vertebrate CNS, the differentiation of specific subclasses of motor neurons are defined by a set of homeodomain transcription factors that are induced or inhibited by a gradient distribution of Sonic hedgehog (Shh; [90]). Cranial motor neurons belonging to the SVE are derived from the so-called p3 domain adjacent to the floor plate, and GSE components are derived from the pMN domain dorsal to the p3 domain in the hindbrain. The p3 and pMN progenitor domains are defined by specific expression of the transcription factor–encoding genes, *Nkx2.2* and *olig2*, respectively (Figure 6). In the spinal cord, unlike in the hindbrain, the p3 domain generates V3 interneurons. In the hindbrain, the SVE nuclei are dorsolaterally positioned, with their axons sprouting dorsally from the dorsal exit point to extend peripherally. In contrast, the somata of somatic nerves are located ventromedially, and their axons sprout ventrally from the hindbrain. The SVE somata that differentiate from the p3 domain migrate dorsally to pass through the somata of GSE neurons during development. Thus, the intramedullar axonal morphology of SAN is similar to that of the SVE neurons. In addition to the progenitor domain, SVE and GSE cranial nerves also have different expression patterns of the genes encoding the Lim-homeodomain (Lim-HD) transcription factors [91,92]. Although the SVE neurons express only *Islet1*, GSE neurons express *Islet2* or *Lhx3* or both in addition to *Islet1* (Figure 6). In our analysis of Lim-HD expression in the chicken, the SAN expresses only *Islet1* without *Islet2* or *Lhx3*, indicating that the Lim-HD code of this nerve is of the SVE type (unpublished data by Tada and Kuratani). However, the Lim-HD expression code may not be sufficient to determine the cranial nerve type if it is necessary for axonogenesis, as seen in the phenotype of spinal motor nerves in an *Lhx3/Lhx4* double-null mutant mouse [89].

A few analyses have been made to elucidate the developmental mechanism behind the peculiar morphogenesis of the SAN. For example, the homeodomain-containing transcription factor *Nkx2.9* is expressed in the ventralmost p3 neural progenitor domain together with *Nkx2.2* during early embryogenesis [93] (Figure 6). These molecules are induced by a gradient of Shh, which diffuses from the floor plate and the notochord. Pabst et al. [93] reported that the *Nkx2.9*-null mutant mouse forms considerably shorter and thinner SAN axons than those in the wild type, although the neural differentiation of the SAN is normal. In addition to the SAN, the IXth and Xth nerves also appeared abnormal in approximately 50% of the mutant embryos [93]. Thus, these authors suggest that *Nkx2.9* has a specific function in the hindbrain as a determinant of SVE precursor cells, including the SAN, contradicting the hypothesis that the SAN originated from GSE nerves.

*Gli2* has been shown to be necessary for the initial extension of axons from SAN cell bodies, and netrin-1 and its receptor-encoding gene, *Dcc*, are also required developmentally for the dorsal migration of the SAN and the dorsally directed extension of SAN axons towards the lateral exit point [94]. Development of the SAN axons is affected by disruption of *UNC5C*, which encodes the UNC5 receptor that facilitates chemorepulsion away from the source of Netrin in both invertebrates and vertebrates [95], as occurs with the IVth nerve (GSE) and the phrenic nerve (spinal motor nerve) axons [96]. *UNC5C* is expressed in the SAN, and the SAN cell bodies migrate away from the ventral midline; in *UNC5C*-null mice, the cell bodies are inappropriately clustered in the ventrolateral spinal cord [95].

**Figure 7:**
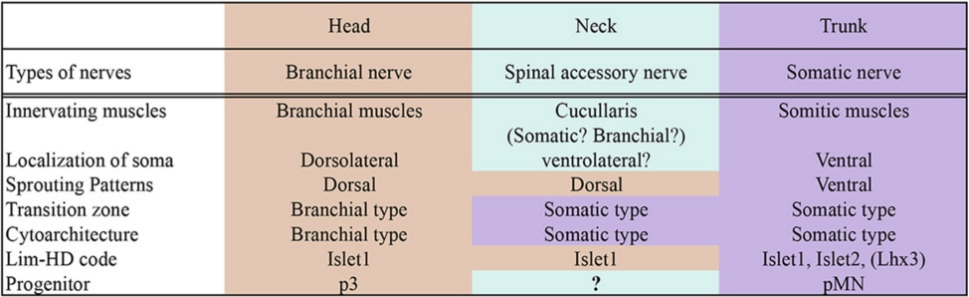
**Summary of head- and trunk-like properties of the SAN in jawed vertebrates.** Note the intermediate nature of the SAN, which is head-like in terms of gene expression and axonal growth pattern but trunk-like in its cytoarchitecture and histological morphology. The developmental and morphological nature of the cucullaris muscle remains controversial.

The above results appear to suggest that the SAN primarily develops as a component of GSE neurons, to which SVE-like axonogenesis was secondarily added through evolution, mainly by assimilating the axonogenetic properties specific to SVE, and presumably by adopting a part of the developmental machinery established for SVE. The GSE axonal pattern also depends on its specific developmental mechanism because the cervical spinal motor neurons also have SAN-like morphological properties, as seen in the phenotype of the mouse lacking the Lim-HD–family genes *Lhx3* and *Lhx4* [89]. Thus, the molecular developmental signatures of the SAN appear to indicate an intermediate or somewhat SVE-like property, and the intermediate sprouting level of the SAN may possibly reflect a default axonal pattern located between the SVE and GSE patterns. The head- and trunk-like properties of the SAN are summarized in Figure 7.

## Conclusions

In this review, we have sought to reconstruct an evolutionary scenario to explain the origin of the SAN, based on the concept that the SAN was originally a GSE nerve, such as that found in the SNP of the lamprey. The hypothetical primitive patterns of the SAN nucleus that we derived from comparative analyses of the gnathostome SANs conspicuously resembles the lamprey SNP, which is positioned in the rostralmost spinal nerve. This scenario predicts that the SAN is a novel structure specific to living gnathostomes that arose through the repatterning of preexisting (SNP-like) spinal motoneurons in the hypothetical ancestor. By de novo upregulation of cranial nerve–specific regulatory genes, the ancestral SAN would have acquired intermediate branchiomeric motoneuron properties, as noted by Benninger and McNeil [12], mainly adopting an SVE-type mechanism to drive axonogenesis and sprouting. Thus, it would not be possible to characterize the accessory nerve based on a simple head/trunk dualism, but it is rather a third category of peripheral nerve. It is conceivable that the gnathostome cucullaris muscle represents a similar intermediate or mixed nature. Although we do not fully understand how such an intermediate anatomical pattern could have evolved, from the ancestral body plan and its developmental program it seems clear that we are looking at something derived in gnathostome evolution, which is neither primitive nor ancestral.

## References

[1] Sambasivan R, Kuratani S, Tajbakhsh S (2011). An eye on the head: the evolution and development of craniofacial muscles. Development.

[2] Romer AS (1972). The vertebrate as a dual animal - somatic and visceral. Evol Biol.

[3] Bass A, Baker R (1991). Evolution of homologous vocal control traits. Brain Behav Evol.

[4] Higashiyama H, Kuratani S (2014). On the maxillary nerve. J Morphol.

[5] Kuratani S (1997). Spatial distribution of postotic crest cells defines the head/trunk interface of the vertebrate body: embryological interpretation of peripheral nerve morphology and evolution of the vertebrate head. Anat Embryol.

[6] Matsuoka T, Ahlberg PE, Kessaris N, Iannarelli P, Dennehy U, Richardson WD, McMahon AP, Köntges G (2005). Neural crest origins of the neck and shoulder. Nature.

[7] Edgewort FH (1935). The Cranial Muscles of Vertebrates.

[8] Kuratani S (2008). Evolutionary developmental studies of cyclostomes and origin of the vertebrate neck. Dev Growth Differ.

[9] Straus WL, Howell AB (1936). The spinal accessory nerve and its musculature. Quart Rev Biol.

[10] Willemse JJ (1958). The innervation of the muscles of the trapezius-complex in giraffe, okapi, camel and llama. Arch Néerland Zoologie.

[11] DeToledo JC, David NJ (2001). Innervation of the sternocleidomastoid and trapezius muscles by the accessory nucleus. J Neuro-Ophthalmol.

[12] Benninger B, McNeil J (2010). Transitional nerve: a new and original classification of a peripheral nerve supported by the nature of the accessory nerve (CN XI). Neurol Res Int.

[13] Edgeworth FH (1911). On the morphology of the cranial muscles in some vertebrates. Quart J microsc Sci.

[14] McKenzie J (1962). The development of the sternomastoid and trapezius muscles. Cont Embryol.

[15] Couly GF, Coltey PM, Le Douarin NM (1993). The triple origin of skull in higher vertebrates: a study in quail-chick chimeras. Development.

[16] Noden DM (1983). The role of the neural crest in patterning of avian cranial skeletal, connective, and muscle tissues. Dev Biol.

[17] Huang R, Zhi Q, Ordahl CP, Christ B (1997). The fate of the first avian somite. Anat Embryol.

[18] Theis S, Patel K, Valasek P, Otto A, Pu Q, Harel I, Tzahor E, Tajbakhsh S, Christ B, Huang R (2010). The occipital lateral plate mesoderm is a novel source for vertebrate neck musculature. Development.

[19] Kobayashi N, Homma S, Okada T, Masuda T, Sato N, Nishiyama K, Sakuma C, Shimada T, Yaginuma H (2013). Elucidation of target muscle and detailed development of dorsal motor neurons in chick embryo spinal cord. J Comp Neurol.

[20] Kanan CV (1969). Spinal accessory nerve of the camel, *Camelus dromedarius*. Acta Anat.

[21] Cui S, Xie ZM (1998). Gross anatomy of the accessory nerve and vagus nerve of the head and cranial neck region in the Bactrian camel. Vet J.

[22] Krammer EB, Lischka MF, Egger TP, Riedl M, Gruber H (1987). The motoneuronal organization of the spinal accessory nuclear complex. Adv Anat Embryol Cell Biol.

[23] Ericsson R, Knight R, Johanson Z (2013). Evolution and development of the vertebrate neck. J Anat.

[24] Diogo R, Ziermann JM (2014). Development of fore- and hindlimb muscles in frogs: morphogenesis, homeotic transformations, digit reduction, and the forelimb-hindlimb enigma. J Exp Zool Mol Dev Evol.

[25] Huang RJ, Zhi QX, Patel K, Wilting J, Christ B (2000). Contribution of single somites to the skeleton and muscles of the occipital and cervical regions in avian embryos. Anat Embryol.

[26] Piekarski N, Olsson L (2007). Muscular derivatives of the cranialmost somites revealed by long-term fate mapping in the Mexican axolotl (*Ambystoma mexicanum*). Evol Dev.

[27] Christ B, Jacob HJ, Jacob M (1974). Origin of wing musculature. Experimental studies on quail and chick embryos. Experientia.

[28] Christ B, Jacob HJ, Jacob M, Wachtler F (1982). On the origin, distribution and determination of avian limb mesenchymal cells. Prog Clin Biol Res.

[29] Epperlein HH, Khattak S, Knapp D, Tanaka EM, Malashichev YB (2012). Neural crest does not contribute to the neck and shoulder in the Axolotl (*Ambystoma mexicanum*). PLoS ONE.

[30] Kague E, Gallagher M, Burke S, Parsons M, Franz-Odendaal T, Fisher S (2012). Skeletogenic fate of zebrafish cranial and trunk neural crest. PLoS ONE.

[31] Kuratani S (2003). Evolutionary developmental biology and vertebrate head segmentation: a perspective from developmental constraint. Theory Biosci.

[32] Couly GF, Coltey PM, Le Douarin NM (1992). The developmental fate of the cephalic mesoderm in quail-chick chimeras. Development.

[33] Adachi N, Takechi M, Hirai T, Kuratani S (2012). Development of the head and trunk mesoderm in the dogfish, Scyliorhinus torazame. II. Comparison of gene expressions between the head mesoderm and somites with reference to the origin of the vertebrate head. Evol Dev.

[34] Addens JL (1933). The motor nuclei and root of the cranial and first spinal nerves of vertebrate Part I. Introduction. Cyclostomes. Z Anat Entwickl-Gesch.

[35] Sperry DG, Boord RL (1993). Organization of the vagus in elasmobranchs: its bearing on a primitive gnathostome condition. Acta Anat.

[36] Szekely G, Matesz C (1988). Topography and organization of cranial nerve nuclei in the sand lizard, *Lacerta agilis*. J Comp Neurol.

[37] Wake DB, Nishikawa KC, Dicke U, Roth G (1988). Organization of the motor nuclei in the cervical spinal cord of salamanders. J Comp Neurol.

[38] Oka Y, Satou M, Ueda K (1987). Morphology and distribution of the motor neurons of the accessory nerve (nXI) in the Japanese toad: a cobaltic lysine study. Brain Res.

[39] Black D (1917). The motor nuclei of the cerebral nerves in phylogeny. A study of the phenomena of neurobiotaxis. II. Amphibia. J Comp Neurol.

[40] Bass A, Gilland EH, Baker R (2008). Evolutionary origins for social vocalization in a vertebrate Hindbrain–spinal compartment. Science.

[41] Windle W (1931). The sensory components of the spinal accessory nerve. J Comp Neurol.

[42] William L, Straus J, Howell A (1936). The spinal accessory nerve and its musculature. Q Rev Biol.

[43] Pearson AA (1937). The spinal accessory nerve in human embryos. J Comp Neurol.

[44] Romanes GJ (1940). The spinal accessory nerve in the sheep. J Anat.

[45] Romanes GJ (1941). The development and significance of the cell columns in the ventral horn of the cervical and upper thoracic spinal cord of the rabbit. J Anat.

[46] McKenzie J (1955). The morphology of the sternomastoid and trapezius muscles. J Anat.

[47] Flieger S (1964). Experimental determination of the site and extension of the nucleus of the accessory nerve (Xi) in sheep. Act Anat.

[48] Flieger S (1966). Experimental demonstration of the position and extent of the N. accressorius (XI) nucleus in the horse. Act Anat.

[49] Flieger S (1967). Experimental determination of the topography and range of the nucleus nervi accessorii (XI) in the cow. J Hirnforsch.

[50] Holomanova A, Benuska J, Durkovicova C, Cierny G, Zlatos J (1973). Localization of the motor cells after denervation of the sternocleidomastoid muscle in the cat. Fol Morphol.

[51] Holomanova A, Cierny G, Zlatos J (1972). Localization of the motor cells of the spinal root of the accessory nerve in the cat. Fol Morphol.

[52] Fahrer H, Ludin HP, Mumenthaler M, Neiger M (1974). The innervation of the trapezius muscle. An electrophysiological study. J Neurol.

[53] Rapoport S (1978). Location of sternocleidomastoid and trapezius motoneurons in the cat. Brain Res.

[54] Vanner SJ, Rose PK (1984). Dendritic distribution of motoneurons innervating the three heads of the trapezius muscle in the cat. J Comp Neurol.

[55] Gottschall J, Zenker W, Neuhuber W, Mysicka A, Muntener M (1980). The sternomastoid muscle of the rat and its innervation. Muscle fiber composition, perikarya and axons of efferent and afferent neurons. Anat Embryol.

[56] Robards MJ, Stritzel M, Robertson RT (1980). Ventral horn cells of the cervical cord project to neck muscles and brain. Brain Res.

[57] Karim MA, Nah SH (1981). Localization of motoneurons innervating the sternocleidomastoid muscle in the monkey, rat and rabbit: a horseradish peroxidase study. Brain Res.

[58] Kitamura S, Sakai A (1982). A study on the localization of the sternocleidomastoid and trapezius motoneurons in the rat by means of the HRP method. Anat Rec.

[59] Augustine JR, White JF (1986). The accessory nerve nucleus in the baboon. Anat Rec.

[60] Jenny A, Smith J, Decker J (1988). Motor organization of the spinal accessory nerve in the monkey. Brain Res.

[61] Berry M, Ibrahim M, Carlile J, Ruge F, Duncan A, Butt AM (1995). Axon-glial relationships in the anterior medullary velum of the adult rat. J Neurocytol.

[62] Lachman N, Acland RD, Rosse C (2002). Anatomical evidence for the absence of a morphologically distinct cranial root of the accessory nerve in man. Clin Anat.

[63] Hayakawa T, Takanaga A, Tanaka K, Maeda S, Seki M (2002). Ultrastructure and synaptic organization of the spinal accessory nucleus of the rat. Anat Embryol.

[64] Ullah M, Salman SS (1986). Localisation of the spinal nucleus of the accessory nerve in the rabbit. J Anat.

[65] Yan J, Aizawa Y, Hitomi J (2007). Localization of motoneurons that extend axons through the ventral rami of cervical nerves to innervate the trapezius muscle: a study using fluorescent dyes and 3D reconstruction method. Clin Anat.

[66] Ueyama T, Satoda T, Tashiro T, Sugimoto T, Matsushima R, Mizuno N (1990). Infrahyoid and accessory motoneurons in the Japanese monkey (*Macaca fuscata*). J Comp Neurol.

[67] Satomi H, Takahashi K, Aoki M, Kasaba T, Kurosawa Y, Otsuka K (1985). Localization of the spinal accessory motoneurons in the cervical cord in connection with the phrenic nucleus: an HRP study in cats. Brain Res.

[68] Clavenzani P, Scapolo PA, Callegari E, Barazzoni AM, Petrosino G, Lucchi ML, Bortolami R (1994). Motoneuron organisation of the muscles of the spinal accessory complex of the sheep investigated with the fluorescent retrograde tracer technique. J Anat.

[69] Ullah M, Mansor O, Ismail ZI, Kapitonova MY, Sirajudeen KN (2007). Localization of the spinal nucleus of accessory nerve in rat: a horseradish peroxidase study. J Anat.

[70] Dubois FS, Foley FJ (1936). Experimental studies on the vagus and spinal accessory nerves in the cat. Anat Rec.

[71] Matesz C, Székely G (1983). The motor nuclei of the glossopharyngeal-vagal and the accessorius nerves in the rat. Act Biol Hung.

[72] Lenhossék M (1890). Ueber Nervenfasern in den hinteren Wurzeln, welche aus dem Vorderhorn entspringen.

[73] Hirsch MR, Glover JC, Dufour HD, Brunet JF, Goridis C (2007). Forced expression of Phox2 homeodomain transcription factors induces a branchio-visceromotor axonal phenotype. Dev Biol.

[74] Kappers A (1919). Phenomena of neurobiotaxis as demonstrated by the position of the motor nuclei of the oblongata. J Nerv Ment Dis.

[75] Kappers A (1921). On structual laws in the nervous system: the principes of neurobiotaxis. Brain.

[76] Nugent SG, O'Sullivan VR, Fraher JP, Rea BB (1991). Central-peripheral transitional zone of the spinal accessory nerve in the rat. J Anat.

[77] Rossiter JP, Fraher JP (1990). Intermingling of central and peripheral nervous tissues in rat dorsolateral vagal rootlet transitional zones. J Neurocytol.

[78] Fraher JP, Kaar GF (1986). The lumbar ventral root-spinal cord transitional zone in the rat. A morphological study during development and at maturity. J Anat.

[79] Fraher JP (1999). The transitional zone and CNS regeneration. J Anat.

[80] Kuraku S, Kuratani S (2006). Timescale for cyclostome evolution inferred with a phylogenetic diagnosis of hagfish and lamprey cDNAs. Zool Sci.

[81] Kuratani S, Kuraku S, Murakami Y (2002). Lamprey as an Evo-Devo model: lessons from comparative embryology and molecular phylogenetics. Genesis.

[82] Kusakabe R, Kuraku S, Kuratani S (2011). Expression and interaction of muscle-related genes in the lamprey imply the evolutionary scenario for vertebrate skeletal muscle, in association with the acquisition of the neck and fins. Dev Biol.

[83] Dietrich S (1999). Regulation of hypaxial muscle development. Cell Tissue Res.

[84] Winslow BB, Takimoto-Kimura R, Burke AC (2007). Global patterning of the vertebrate mesoderm. Dev Dyn.

[85] Trinajstic K, Sanchez S, Dupret V, Tafforeau P, Long J, Young G, Senden T, Boisvert C, Power N, Ahlberg PE (2013). Fossil musculature of the most primitive jawed vertebrates. Science.

[86] Tahara Y (1988). Normal stages of development in the lamprey, *Lampetra reissneri* (Dybowski). Zool Sci.

[87] Murakami Y, Ogasawara M, Sugahara F, Hirano S, Satoh N, Kuratani S (2001). Identification and expression of the lamprey *Pax6* gene: evolutionary origin of the segmented brain of vertebrates. Development.

[88] Glover JC (1995). Retrograde and anterograde axonal tracing with fluorescent dextran amines in the embryonic nervous system. Neurosci Protocols.

[89] Sharma K, Sheng HZ, Lettieri K, Li H, Karavanov A, Potter S, Westphal H, Pfaff SL (1998). LIM homeodomain factors Lhx3 and Lhx4 assign subtype identities for motor neurons. Cell.

[90] Briscoe J, Pierani A, Jessell TM, Ericson J (2000). A homeodomain protein code specifies progenitor cell identity and neuronal fate in the ventral neural tube. Cell.

[91] Varela-Echavarria A, Pfaff SL, Guthrie S (1996). Differential expression of LIM homeobox genes among motor neuron subpopulations in the developing chick brain stem. Mol Cell Neurosci.

[92] Guthrie S (2007). Patterning and axon guidance of cranial motor neurons. Nat Rev Neurosci.

[93] Pabst O, Rummelies J, Winter B, Arnold HH (2003). Targeted disruption of the homeobox gene Nkx2.9 reveals a role in development of the spinal accessory nerve. Development.

[94] Dillon AK, Fujita SC, Matise MP, Jarjour AA, Kennedy TE, Kollmus H, Arnold HH, Weiner JA, Sanes JR, Kaprielian Z (2005). Molecular control of spinal accessory motor neuron/axon development in the mouse spinal cord. J Neurosci.

[95] Dillon AK, Jevince AR, Hinck L, Ackerman SL, Lu X, Tessier-Lavigne M, Kaprielian Z (2007). UNC5C is required for spinal accessory motor neuron development. Mol Cell Neurosci.

[96] Burgess RW, Jucius TJ, Ackerman SL (2006). Motor axon guidance of the mammalian trochlear and phrenic nerves: Dependence on the netrin receptor *Unc5c* and modifier loci. J Neurosci.

